# Missouri State Medical Directory

**Published:** 1893-09

**Authors:** 


MISSOURI STATE MEDICAL DIRECTORY, Containing a careful revised list of Physicians, Dentists and Druggists, together with the Colleges, Hospitals, Societies, and Medical Journals of the State, arranged by counties for convenience of Society Secretaries. Pocket size, 120 pp., cloth, gold embossing. Published by the Medical Fortnightly, 1006 Olive Street, St. Louis. Price, $3, postpaid.

